# ITLN1 exacerbates Crohn's colitis by driving ZBP1-dependent PANoptosis in intestinal epithelial cells through antagonizing TRIM8-mediated CAPN2 ubiquitination

**DOI:** 10.7150/ijbs.105550

**Published:** 2025-05-31

**Authors:** Jie Zhao, Yujiang Li, Pu Ying, Yan Zhou, Ziwei Xu, Dongmei Wang, Honggang Wang, Liming Tang

**Affiliations:** 1 Department of Gastrointestinal Surgery, The Affiliated Changzhou No. 2 People's Hospital of Nanjing Medical University, The Third Affiliated Hospital of Nanjing Medical University, Changzhou Medical Center, Nanjing Medical University, China.; 2 Department of Gastroenterology, The Affiliated Taizhou People's Hospital of Nanjing Medical University, Taizhou School of Clinical Medicine, Nanjing Medical University, China.; 3 Department of Orthopedics, Changshu Hospital Affiliated to Nanjing University of Chinese Medicine, China.; 4 Department of General Surgery, The First Affiliated Hospital of Nanjing Medical University, China.; 5 Department of General Surgery, Affiliated Taizhou People's Hospital of Nanjing Medical University, Taizhou School of Clinical Medicine, Nanjing Medical University, China.

**Keywords:** Crohn's disease, ITLN1/TRIM8/CAPN2 axis, PANoptosis, ubiquitination

## Abstract

**Background:** This study aimed to investigate the mechanisms by which PANoptosis of intestinal epithelial cells (IECs) promotes Crohn's disease (CD) progression.

**Methods:** Single-cell RNA sequencing (scRNA-seq) was performed on inflamed and uninflamed colon tissues from patients with CD. The biological functions of intelectin-1 (ITLN1) in inflammation and PANoptosis were verified through *in vitro* experiments. The molecular mechanisms underlying its biological functions were examined using co-immunoprecipitation (Co-IP) combined with mass spectrometry (MS) and RNA-seq and further validated with rescue experiments. Additionally, the *in vivo* function of ITLN1 regulation on inflammation, PANoptosis, and the intestinal mucosal barrier was explored in interleukin-10 knockout (IL-10 KO) colitis model mice.

**Results:** ITLN1 was significantly overexpressed in IECs from inflamed colon tissues and specifically associated with CD-related inflammatory markers. RNA-seq and *in vitro* experiments indicated that ITLN1 promotes inflammation, PANoptosis, and impaired tight junctions. Co-IP and MS analyses revealed that ITLN1 can bind to the PANoptosis-promoting protein calpain-2 (CAPN2) and enhance its stability. The E3 ubiquitin ligase, a tripartite motif containing 8 (TRIM8), directly interacts with CAPN2 and mediates its ubiquitination degradation. ITLN1 can bind to TRIM8, and its impact on inflammation and Z-DNA binding protein 1 (ZBP1)-induced PANoptosis can be antagonized by CAPN2. These *in vivo* studies indicated that short hairpin-ITLN1 improves colonic inflammation and intestinal barrier function in IL-10 KO mice.

**Conclusion:** We identified the ITLN1-TRIM8-CAPN2 axis that drives IEC PANoptosis in CD progression. Pharmacological inhibition of ITLN1 significantly mitigated epithelial damage and colitis both *in vivo* and* in vitro*, establishing ITLN1-targeted therapies and PANoptosis modulation as viable clinical strategies for CD treatment.

## Introduction

Crohn's disease (CD) is a persistent inflammatory condition characterized by autoimmune dysregulation. As one of the principal forms of inflammatory bowel disease (IBD), this disorder is characterized by pan-enteric tropism, potentially involving any segment from the oral cavity to the anal canal 1. CD poses a significant global public health challenge, with a notable incidence worldwide 1. Incidence rates are exceptionally high in Western countries compared with Asian regions 2. However, recent data indicate an increasing trend in the incidence of CD in China, especially in major cities 3. The complex pathogenesis of CD involves genetics, immunity, and environmental factors and remains unclear 4. The CD is currently clinically incurable and prone to recurrence, primarily relying on nutritional support, medication, and surgical interventions to alleviate symptoms and control inflammation 5,6. This underscores the imperative for mechanistic investigations to uncover novel molecular targets for improved clinical interventions.

Emerging evidence implicates intestinal epithelial cells (IECs) dyshomeostasis as a pivotal contributor to CD pathophysiology 7. Pathogenic cascades involve multidimensional disruptions: (1) malabsorption of essential ions and metabolites; (2) compromised mucosal integrity through junctional complex degradation; (3) ecological shifts in commensal microbiota; (4) systemic immune network destabilization 8,9. A reduction in the number of IECs and the destruction of tight junctions are frequently observed in CD, resulting in impaired intestinal epithelium mechanical barrier function 10. This impairment permits many bacteria and other pathogenic factors to penetrate the epithelial layer and directly interact with the intestinal immune system, leading to a persistently and excessively activated immune response and promoting CD progression 11.

PANoptosis, a novel inflammatory cell death paradigm regulated by the PANoptosome multiprotein complex, synthesizes molecular hallmarks of pyroptosis, apoptosis, and necroptosis, transcending singular classification within conventional death modalities 12,13. This coordinated demise mechanism functions as a molecular amplifier of inflammation, orchestrating the secretion of cytokine storm initiators that propagate pathological immune activation—a critical driver of autoimmune pathogenesis 14. Current investigations have delineated its mechanistic involvement in immune dysregulation disorders, with robust evidence in rheumatoid arthritis (RA) 15 and systemic lupus erythematosus 16. However, to date, no reports have directly linked PANoptosis with CD.

Single-cell sequencing demonstrated that ITLN1 overexpression in colonic epithelium is associated with inflammatory severity. Our functional studies demonstrated that ITLN1 facilitates PANoptosis by stabilizing CAPN2 through competition with TRIM8, thereby establishing a mechanistic connection between PANoptosis and CD progression. This axis represents a new therapeutic target, as evidenced by ITLN1 inhibition, which mitigates epithelial damage in both *in vivo* and *in vitro* models. The ITLN1-CAPN2 interaction interface offers a framework for creating biologics to inhibit pathogenic PANoptosis, addressing a critical requirement in CD precision therapeutics.

## Materials and methods

### Patient samples

Colonic tissue samples (both inflamed and non-inflamed regions) were obtained from 14 CD patients undergoing colectomy or ileocolectomy. All procedures strictly complied with the ethical guidelines outlined in the Helsinki Declaration. Immediately after resection, lamina propria-containing tissue fragments (approximately soybean-sized) were flash-frozen in liquid nitrogen and stored in an ultra-low temperature freezer at -80°C for subsequent RNA extraction. This prospective, single-center observational study was conducted at the First Affiliated Hospital of Nanjing Medical University (2023-2024) with approval from the Institutional Ethics Committee (Approval No.: 2023-SR-115). The study participants' inclusion and exclusion criteria are outlined in Table 1. All participants provided informed consent. Table 2 presents the demographic data of the enrolled patients, including age, gender, disease, and location. Before surgery, all enrolled patients with CD were assessed for inflammatory markers (for instance, C-reactive protein) based on previously reported methods 17,18.

### Single-cell RNA (scRNA) sequencing

ScRNA-seq and bioinformatic analysis were conducted by Genechem Co., Ltd. Sequencing libraries were generated using the BD Rhapsody WTA Pipeline (version 1.8). Raw FASTQ files were aligned to reference genomes (human GRCh38/mouse mm10) with transcriptome annotations (GENCODE version 32/Ensembl 98 for human; GENCODE version M23/Ensembl 98 for mouse). A UMI count matrix was processed through the Scanpy Python package (version 1.8). Quality control steps included the following: (1) Filtering cells with low UMI/gene counts (below set thresholds); (2) removing cells exhibiting > 10% mitochondrial gene expression. Post-QC, data underwent library size normalization (normalize_total function) and log transformation. Highly variable genes were identified using the methods described by Macosko et al. 19. Dimensionality reduction via PCA was followed by graph-based clustering and 2D/3D Uniform Manifold Approximation and Projection (UMAP) visualization. Cluster-specific marker genes were determined using Seurat's Wilcoxon test (adjusted *P* < 0.05, |log2FC| > 2) 20. Functional enrichment analysis (GO/KEGG pathways) was performed with g:Profiler2 21 using hypergeometric testing.

### Co-immunoprecipitation (Co-IP) and protein mass spectrometry (MS)

Cells were lysed with IP lysis buffer, and total proteins were isolated via centrifugation. After verifying protein quality using Western blotting, Co-IP experiments proceeded with SureBeads magnetic beads. Resuspended beads (20 μL) were washed with phosphate buffer saline (PBS) with Tween 20, incubated with 5 μL IP antibody on a rotary shaker (1 h, RT), and subsequently mixed with lysate-containing target proteins (2 h, RT). Post-washing, protein complexes were denatured in a loading buffer (40 μL, 95 °C, and 10 min) and collected for Western blotting or MS.

Silver staining was performed using the Fast Silver Stain Kit (Beyotime). Samples were analyzed via an UltiMate 3000 RSLCnano system coupled to a Q Exactive HF MS (Thermo). Raw MS data were processed with MaxQuant (version 1.6.6.0) 22, aligned to the UniProt human database (2023-06-19 release, 20,423 entries) 23. Significant hits required a fold change > 2 and ≥ 2 unique peptides.

### Animal experiments

C57BL/6 IL-10 knockout (KO) mice (GemPharmatech Co. Ltd.) were housed under specific pathogen-free conditions and used as colitis models. Twelve-week-old male wild-type (WT) and IL-10 knockout (KO) mice were divided into four groups (n = 5 per group): (i) WT group; (ii) IL-10 KO mice treated with normal saline; (iii) IL-10 KO mice treated with a short hairpin (sh)-ITLN1 (administered 200 μL of 1e11 viral genomes adeno-associated virus (AAV) vector carrying sh-ITLN1 via enema once); (iv) IL-10 KO mice were treated with sh-ITLN1 combined with oe-CAPN2 (AAV carried sh-ITLN1 and oe-CAPN2 via enema once). Mice were euthanized via cervical dislocation one-week post-intervention, and proximal colon tissues were collected for analysis. All procedures were approved by the Ethics Committee of Nantong University (No. S20200323-289).

### Disease activity index (DAI)

Based on standardized criteria, a score of 1 was allocated for each observed clinical manifestation: unkempt fur, fecal occult blood, minor rectal prolapse (< 1 mm), or loose stool consistency 24. Severe diarrhea or rectal prolapse exceeding 1 mm warranted an additional point. The final DAI was calculated as the cumulative sum of these scores.

### Histological analysis

Following euthanasia, colon length was recorded, and proximal colon segments were collected. Tissues were washed with PBS, preserved in 10% formalin, and paraffin-embedded. Sections (6 μm) were prepared for hematoxylin-eosin (HE) or Alcian Blue-Periodic Acid-Schiff (AB-PAS) staining. Two blinded pathologists independently assessed colitis severity and inflammation using established grading guidelines 25.

### Determination of levels of pro-inflammatory cytokines

IL-17, tumor necrosis factor-alpha (TNF-α), and interferon-gamma (IFN-γ) levels in proximal colon homogenates were analyzed via ELISA (R&D Systems). Briefly, tissues were rinsed, homogenized in PBS, and centrifuged (5,000 ×g, 5 min). Supernatants (50 μL/well) were loaded onto 96-well plates with Assay Diluent RDI-63. After incubation and washes, absorbance at 450 nm was quantified using a BioTek EL800 reader.

### Epithelial apoptosis analysis

Epithelial apoptosis was evaluated via TUNEL assay (In Situ Cell Death Detection Kit, Roche) following standard protocols 26. Colon sections underwent permeabilization with 1% Triton X-100 and 0.1% sodium citrate, followed by incubation with TUNEL reagent in darkness. Nuclei were counterstained with DAPI (Servicebio), and slides were mounted in 50% glycerol. TUNEL-positive cells were quantified per field using confocal microscopy (Olympus).

### Epithelial cells isolation

Colonic epithelial cells from human and murine tissues were isolated via chelation-based methods 27. Murine colon segments (0.5 cm) or surgically resected human CD specimens (inflamed/uninflamed) were rinsed in cold PBS to clear debris. Tissues were treated with dithiothreitol (2 mmol/L) and EDTA (1 mmol/L) in PBS under gentle agitation (37 °C, 20 min, two cycles). Cell suspensions were purified via percoll-RPMI density gradient centrifugation (200 × *g*, 5 min), and pelleted epithelial cells were retained for downstream applications.

### Immunofluorescence staining of colon tissues and cells

Immunofluorescence staining of colon tissues or cells was conducted to investigate protein localization and expression levels, as described by a previous study 28. Cell coverslips or frozen sections were rinsed, followed by antigen retrieval using EDTA buffer in a microwave. After washing, the sections were dried, circled with a hydrophobic barrier pen, and soaked in 3% hydrogen peroxide to remove endogenous peroxidase. After washing again in PBS, BSA was added to the block for 30 min. The primary antibody was applied and incubated overnight at 4 °C. The sections were subsequently treated with an HRP-conjugated secondary antibody at room temperature for 50 min, washed, and CY3-TSA was added and incubated in the dark for 10 min. This process was repeated for subsequent primary and secondary antibodies with FITC-TSA, CY5-TSA, and 594-TSA. Nuclei were counterstained with DAPI, and the slides were mounted and photographed. The primary antibodies used included anti-ZO-1 (Abcam, #ab221547), anti-occludin (Abcam, #ab216327), anti-ZBP1 (Thermo Fisher, #PA5-20455), ITLN1 (Proteintech, #11770-1-AP), anti-caspase1 (Servicebio, #GB11383), anti-RIPK3 (Servicebio, #GB115458), anti-ASC (Servicebio, #GB113966, GB115270), and anti-TRIM8 (Proteintech, #27463-1-AP).

### Western blotting

Protein lysates from tissues or cells were prepared using RIPA buffer (Beyotime) supplemented with phosphatase/protease inhibitors. Protein concentration was quantified via BCA assay (Pierce Biotechnology). As described by a previous study 29, equal amounts (15 µg) were resolved on 10% SDS-PAGE gels and transferred to PVDF membranes (Millipore). After blocking with 5% non-fat milk, membranes were probed with primary antibodies at 4 °C overnight, followed by secondary antibody incubation (2 h). Signals were developed using chemiluminescent reagents (Cell Signaling Technology) and analyzed with ImageJ. Loading controls included GAPDH, vinculin, or β-actin. Primary antibodies were as follows: anti-ZO-1 (Abcam, #ab221547), anti-occludin (Abcam, #ab216327), anti-CAPN2 (Abcam, #ab236650), anti-caspase1 (AdipoGen, #AG-20B-0042), anti-caspase3 (CST, #9662), anti-caspase-7 (CST, #9492), anti-pMLKL (CST, #37333), anti-MLKL (Servicebio, #GB115699), anti-RIPK3 (Servicebio, #GB115458), anti-pRIPK3 (CST, #91702), anti-GSDMD-N (Abcam, #ab215203), anti-ASC (Servicebio, #GB113966, GB115270), anti-TRIM8 (Proteintech, #27463-1-AP), anti-Ub-WT (Servicebio, #GB115700), anti-Ub-K48 (Abcam, #ab140601), and anti-Ub-K63 (Abcam, #ab179434).

### RNA extraction and qRT-PCR

Total RNAs were extracted from colon tissues/cells using TRIzol reagent (Invitrogen, Carlsbad, CA, USA) following the manufacturer's protocol. The extracted RNA was reverse transcribed into cDNA using the First-Strand cDNA Synthesis Kit (Invitrogen). Furthermore, qPCR was performed using SYBR Green (Applied Biosystems) on an ABI 7500 system. Relative expression levels were normalized via the 2^-△△Ct^ method, with triplicate technical replicates. Primer sequences are detailed in Table 3.

### Transmission electron microscopy (TEM) of tight junctions (TJs)

TJs were analyzed via TEM, as described previously 30. Tissue samples were fixed in 4% glutaraldehyde, post-fixed in 1% osmium tetroxide, dehydrated, and embedded in Epon 812 resin. Ultrathin sections were stained with uranyl acetate and lead citrate and imaged using a Hitachi H-600 TEM.

### Ussing chamber studies

Proximal colon segments were mounted in Lucite chambers with Ringer's buffer (37 °C) to evaluate permeability 31. Basal mannitol flux was measured by adding 1 mM mannitol to the mucosal compartment. Transepithelial electrical resistance (TEER) and short-circuit current (Isc) were recorded via an automated voltage clamp, with TEER calculated using Ohm's law 32.

### Intestinal permeability assay

*In vivo* intestinal permeability was assessed using FITC-dextran (60 mg/100 g body weight; Sigma-Aldrich) administered via oral gavage 33. The serum was collected 4 h post-administration and was analyzed for FITC-dextran levels via fluorescence quantification (BMG Labtech).

### Flow cytometry (FCM) of apoptosis

Epithelial cell apoptosis was assessed using propidium iodide (PI) and Annexin V-FITC staining, following a previously described method 34. Cells were stained with 10 µL PI and 5 µL Annexin V-FITC and analyzed with an EpicsAltra flow cytometer (Beckman Coulter). Early apoptotic cells (Annexin V+/PI-) and late apoptotic cells (Annexin V+/PI+) were quantified with triplicate technical replicates.

### Cell transfection

NCM460 cells were transfected with TRIM8 siRNA (Lipofectamine™2000, Thermo Fisher), ITLN1 shRNA (pLKO.1 vector, Sangon Biotech), or TRIM8/CAPN2 overexpression plasmids (pcDNA3.1), following standard protocols. siRNA/shRNA sequences are listed in Table 4.

### Detection of ubiquitination levels

To detect the ubiquitination levels of CAPN2, HEK 293 cells were transfected with CAPN2 to promote its overexpression. After 24 h, CAPN2 protein was purified and separated using SDS-PAGE. An anti-ubiquitin antibody was subsequently used to identify which lysine residues in CAPN2 were more prone to ubiquitination. In an *in vitro* reaction, the target protein CAPN2 was mixed with HA-ubiquitin and TRIM8 (a protein hypothesized to induce CAPN2 ubiquitination) in a reaction buffer and incubated. The resulting mixture was analyzed using immunoprecipitation and Western blotting techniques to further study CAPN2 ubiquitination.

### Cycloheximide (CHX) chase assay

HEK293 cells transfected with MG132, si-TRIM8, oe-TRIM8, or controls were treated with CHX (100 μg/mL) 24 h post-transfection 35. CAPN2 protein degradation was monitored via Western blotting at designated timepoints.

### Cell live/dead (Calcein/PI) staining

Live/dead (Calcein/PI) staining was used to assess the proportion of dead cells among IECs, following the manufacturer's instructions (Proteintech, #PF00007). A mixture of 30 µL of 1.5 mM PI and 5 µL of 4 mM Calcein AM was combined with 10 mL of PBS. Cells (5 × 10^5^ /mL) were incubated with a staining solution (vortexed in PBS) for 15 min in the dark and visualized under a fluorescence microscope.

### Statistical analysis

Data were analyzed using SPSS 19.0 or GraphPad Prism 8.0. Group comparisons were conducted using Student's *t*-test or analysis of variance (ANOVA), while Pearson's correlation was used to assesse ITLN1 expression and inflammatory markers in Crohn's disease. Significance was set at *P* < 0.05.

## Results

### IECs were significantly reduced in CD inflammatory tissues

**Figure 1:**
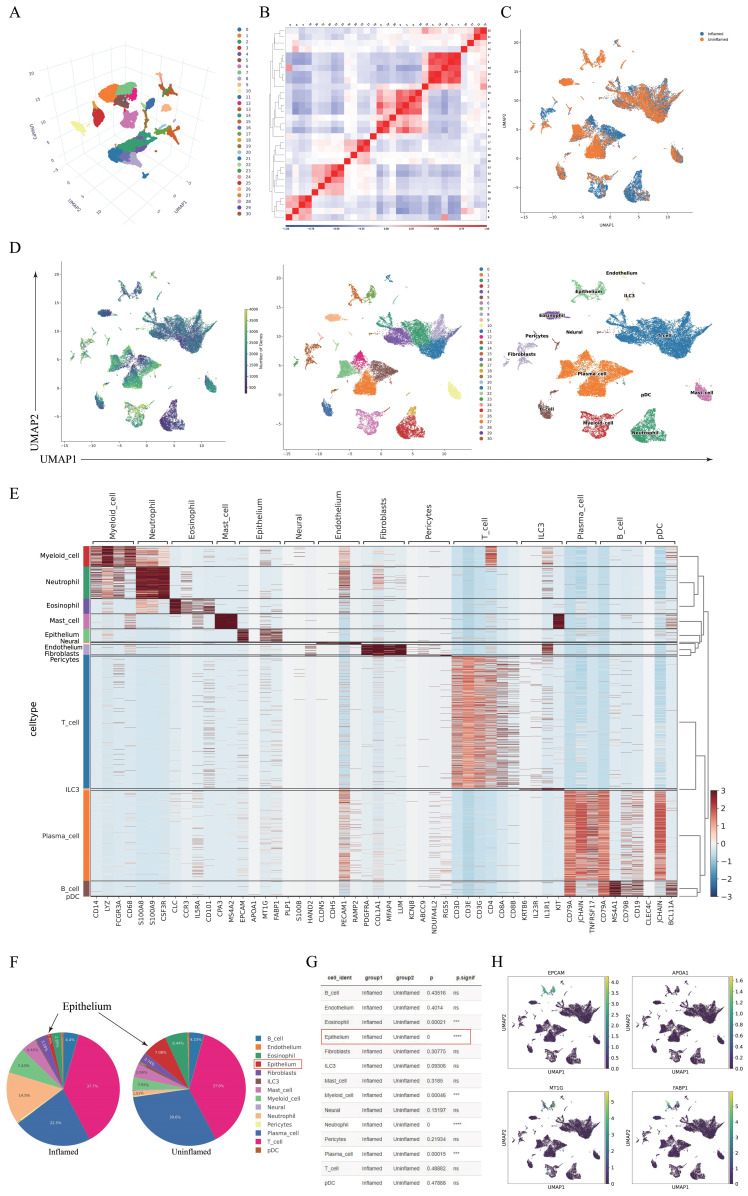
Single-cell RNA sequencing analysis of inflamed and uninflamed colon tissues from patients with CD. (A) Three-dimensional projection of cell clustering in the colon was performed using the UMAP method. (B) Heatmap illustrating the correlation between different cell populations, calculated by Pearson's correlation coefficients. (C) Clustering analysis of cells derived from inflamed and uninflamed colon tissues, highlighting distinct cell groupings. (D) UMAP-based cell type annotation, illustrating various cell types in the colon tissue samples. (E) Expression levels of marker genes across each cell cluster. (F-G) Comparative analysis of cell subgroup distributions between inflamed and uninflamed tissues, indicating statistical significance. (H) Expression profiles of specific IEC marker genes across various cell clusters. IECs, intestinal epithelial cells; CD, Crohn's disease; UMAP, Uniform Manifold Approximation and Projection.

Single-cell RNA sequencing (scRNA-seq) was conducted on colon tissues from patients with CD, including four inflamed and three non-inflamed samples (one excluded due to quality control). Using the UMAP method, we clustered the colon cell populations. The clustering and 3D projection results are illustrated in Figure 1A. Pearson's correlation heatmap (Figure 1B) and cluster distribution plots (Figure 1C) highlighted intergroup heterogeneity. Cell subtypes were annotated using marker gene expression (Figures 1D-E). The comparison of cell subgroup numbers between groups indicated that the reduction in the number of IECs was most significant in the inflamed regions (*P =* 0.000, Figures 1F-G). Given the pivotal role of IECs in CD pathogenesis and their marked depletion in inflamed tissues, subsequent analyses focused on this population. Marker genes (EPCAM, APOA1, MT1G, and FABP1) exhibited cluster-specific expression patterns (Figure 1H).

### The presence of PANoptosis in IECs under inflammatory conditions

**Figure 2:**
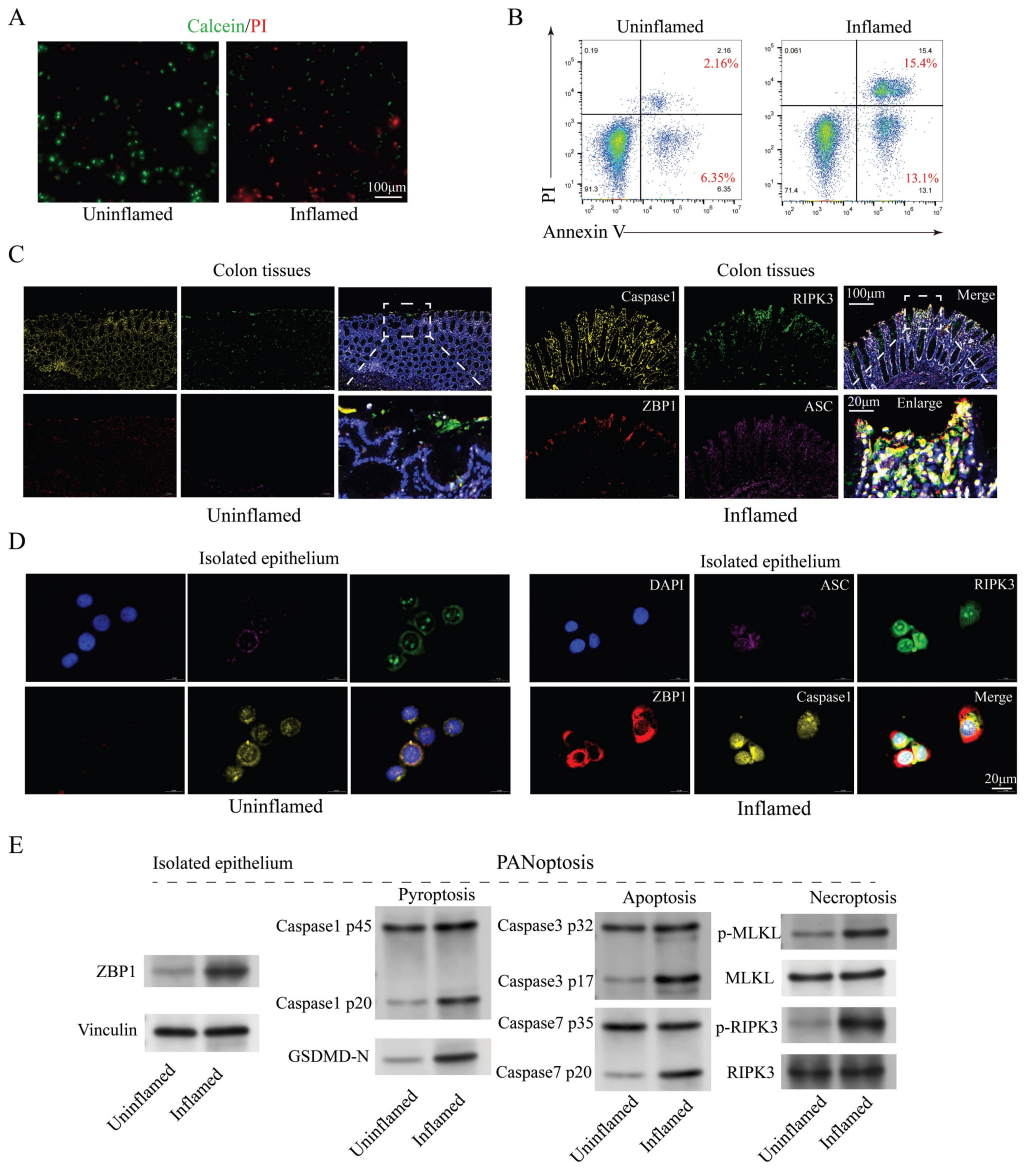
Evidence of PANoptosis in CD under inflammatory conditions. (A) Live/dead cell staining (Calcein/PI) of primary isolated IECs from inflamed and uninflamed colon tissues, illustrating viable (green) and dead (red) cells. Scale bar: 100 μm. (B) FCM analysis of apoptosis with Annexin V/PI staining in primary isolated IECs from inflamed and uninflamed colon tissues. (C) Immunofluorescence analysis of PANoptosis-related marker genes (caspase1, RIPK3, ZBP1, and ASC) in inflamed versus uninflamed regions of colon tissues. Scale bars: 100 and 20 μm. (D) Immunofluorescence detection of PANoptosis-related marker genes (caspase1, RIPK3, ZBP1, and ASC) in isolated IECs from inflamed and uninflamed colon tissues. Scale bar: 20 μm. (E) Western blotting analysis for PANoptosis-related marker genes (caspase1, caspase3, caspase7, GSDMD-N, MLKL, and RIPK3) in isolated IECs from inflamed and uninflamed colon tissues. IECs, intestinal epithelial cells; CD, Crohn's disease; FCM, flow cytometry.

The number of IECs is influenced by various factors, including genetics, lifestyle, inflammation, environment, and medication 36. Considering that this study compares the differences in the number of IECs between inflamed and non-inflamed regions within the same patient with CD, we can exclude these external factors. Inflammation is probably the primary cause of the significant reduction in IEC numbers. The live/dead cell staining (Figure 2A) and apoptosis flow cytometry (Figure 2B) analysis of the primary isolated IECs from colon tissues revealed markedly elevated cell death in inflamed regions, suggesting apoptosis as a key driver of IEC depletion. To delineate the modes of IEC death, markers of apoptosis (caspase3 and caspase7), pyroptosis (ASC, caspase1, and GSDMD-N), and necroptosis (pMLKL and pRIPK3) were analyzed. Western blotting demonstrated significant upregulation of these markers in inflamed colon tissues (Figure 2C) and isolated epithelial cells (Figures 2D-E), implicating multiple forms of cell death, known as PANoptosis, were present in CD inflammation. Additionally, the subcellular localization of these cell death marker proteins in IEC exhibited significant overlap (Figure 2D), indicating the possible formation of protein complexes termed PANoptosomes. These findings suggest that PANoptosis is present in IECs under CD inflammation and may play a role in CD progression.

### ITLN1 in IECs is closely linked to CD inflammation

**Figure 3:**
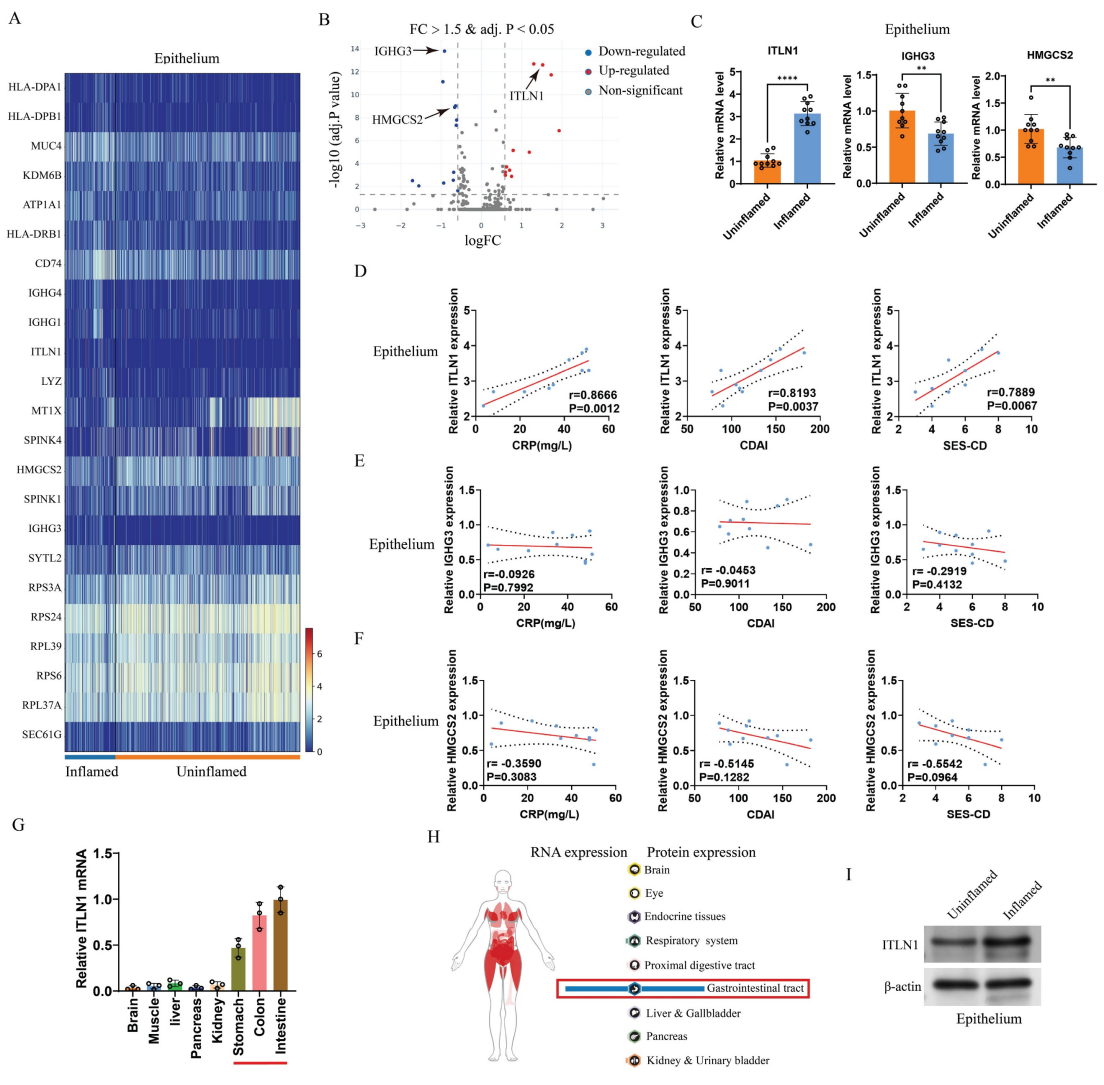
ITLN1 in IECs closely correlates with CD inflammation. (A) Heatmap and (B) volcano plot displaying differentially expressed genes in IECs from inflamed and uninflamed colon tissues of patients with CD (threshold: FC > 1.5 and adjusted *P* value < 0.05). (C) qRT-PCR validation of differentially expressed genes in the isolated epithelium (10 inflamed versus 10 uninflamed), confirming the upregulation of ITLN1, IGHG3, and HMGCS2 in the inflamed epithelium. (D-F) Correlation analysis between ITLN1, IGHG3, and HMGCS2 expression in inflamed epithelium with CD-associated inflammatory markers (CRP, CDAI, and SES-CD), demonstrating significant associations. (G) Relative mRNA expression of ITLN1 in various WT mice tissues, exhibiting its highest expression in the gastrointestinal system. (H) RNA and protein expression of ITLN1 in human tissues, as provided by the Atlas online database. (I) Western blotting analysis of ITLN1 protein expression in IECs from inflamed and uninflamed colon tissues of patients with CD, highlighting the elevated expression in inflamed IECs. CD, Crohn's disease; IECs, intestinal epithelial cells; ITLN1, intelectin-1; FC, fold change; qRT-PCR, quantitative real-time-polymerase chain reaction; CRP, C-reactive protein; CDAI, Crohn's disease activity index; SES-CD, simple endoscopic score for Crohn's disease. Data are presented as mean ± standard deviation (SD). ** *P <* 0.01, *** *P <* 0.001, **** *P <* 0.0001 by paired *t* test.

scRNA-seq analysis identified differentially expressed genes (DEGs) between inflamed and non-inflamed IECs (Figures 3A-B). The top five upregulated and downregulated genes were selected for validation with an expanded sample size (10 inflamed versus 10 uninflamed). Three differentially expressed genes (ITLN1, IGHG3, and HMGCS2) were identified (Figure 3C). The correlation between these three genes and CD-specific inflammatory markers identified ITLN1 as a core molecule (Figures 3D-F). ITLN1 is a protein that contains a carbohydrate-binding domain and can bind to specific carbohydrate structures, and it can be secreted by IECs 37. We examined ITLN1 expression in various tissues (brain, liver, kidney, and gastrointestinal tract). We found that ITLN1 is specifically expressed in the gastrointestinal system, with very low expression in other systems (Figure 3G), consistent with the Atlas database (Figure 3H). Protein expression analysis further confirmed pronounced ITLN1 upregulation in inflamed IECs (Figure 3I). These findings suggest that ITLN1 is probably an important regulatory molecule in the progression of CD inflammation.

### ITLN1 influences PANoptosis in IECs

**Figure 4:**
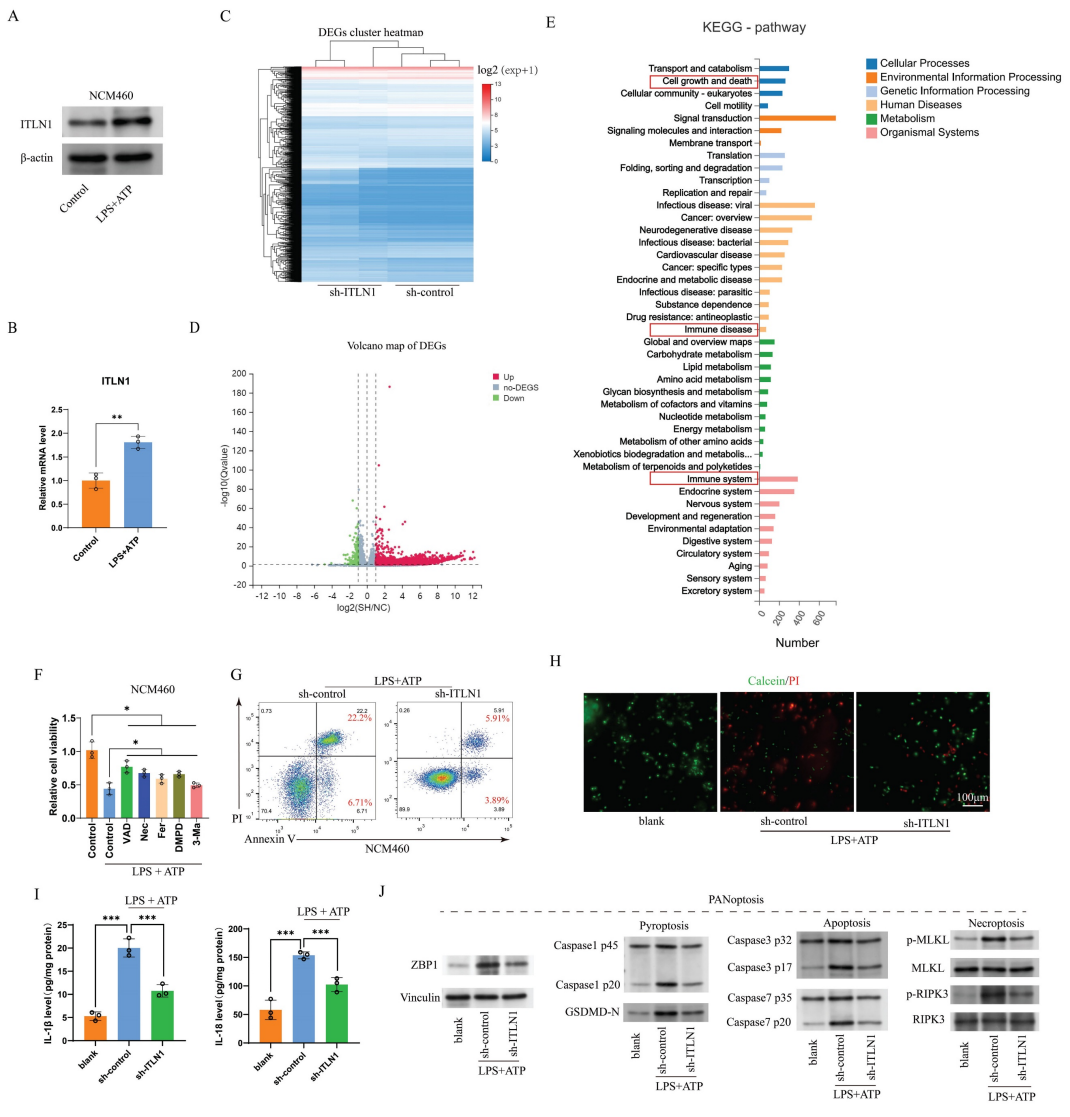
ITLN1 influences PANoptosis in IECs. (A) Western blotting and (B) q-PCR analysis revealing ITLN1 expressions in NCM460 cells with or without LPS/ATP stimulation. (C-D) The heatmap and volcano plots illustrating differentially expressed genes from RNA-seq analysis comparing sh-ITLN1 versus sh-control. (E) KEGG pathway enrichment analysis of differentially expressed genes. (F) Effect of various cell death inhibitors on cell viability in LPS/ATP-induced NCM460 cells. (G) Annexin V/PI FCM analysis of apoptosis in LPS/ATP-induced NCM460 cells treated with sh-ITLN1 or control. (H) Live/dead cell staining (Calcein/PI) of LPS/ATP-induced NCM460 cells, visualizing viable (green) and dead (red) cells. Scale bar: 100 μm. (I) Influence of sh-ITLN1 or control on the proinflammatory cytokine levels in LPS/ATP-induced NCM460 cells. (J) Western blotting analysis for PANoptosis-related marker genes (caspase-1, caspase-3, GSDMD-N, MLKL, RIPK3) in LPS/ATP-induced NCM460 cells. ITLN1, Intelectin-1; IECs, intestinal epithelial cells; LPS, lipopolysaccharide; ATP, adenosine triphosphate; FCM, flow cytometry. ** *P <* 0.01, *** *P <* 0.001, **** *P <* 0.0001 by paired *t* test.

In the lipopolysaccharides (LPS) and adenosine triphosphate (ATP) co-stimulated normal colon mucosa 460 (NCM460) *in vitro* inflammation model, ITLN1 expression was significantly upregulated (Figure 4A), suggesting a close relationship between ITLN1 and inflammation. Three distinct shRNA sequences targeting ITLN1 were designed to investigate ITLN1's functional role. The qRT-PCR and Western blotting (Supplemental Figures 1A-B) identified sh-ITLN1-1 as the most effective construct, selected for downstream analyses. The heatmap and volcano plot of differentially expressed genes from the RNA-seq analysis of sh-ITLN1 and sh-control are depicted in Figures 4B-C, respectively. KEGG pathway enrichment highlighted “cell growth and death” as a top-ranked pathway (Figure 4D), aligning with ITLN1's potential regulatory role in inflammatory cell death. In the LPS/ATP-induced *in vitro* inflammation model, no single death inhibitor could completely reverse the inflammation-induced reduction in cell proliferation activity (Figure 4E), further supporting the existence of PANoptosis. Results of apoptosis FCM (Figure 4F) live/dead (Calcein/PI) staining (Figure 4G) confirmed that sh-ITLN1 inhibited cell death. Furthermore, the proinflammatory cytokines IL-1β and IL-18, which were elevated by LPS + ATP induction, were significantly reduced under the sh-ITLN1 intervention (Figure 4H). Moreover, sh-ITLN1 significantly suppressed the expression of PANoptosis-related marker proteins (Figure 4I). These results further support that ZBP1-induced PANoptosis is probably involved in the progression of CD inflammation, and sh-ITLN1 can inhibit inflammation and ZBP1-induced PANoptosis.

### ITLN1 influences tight junctions in IECs

**Figure 5:**
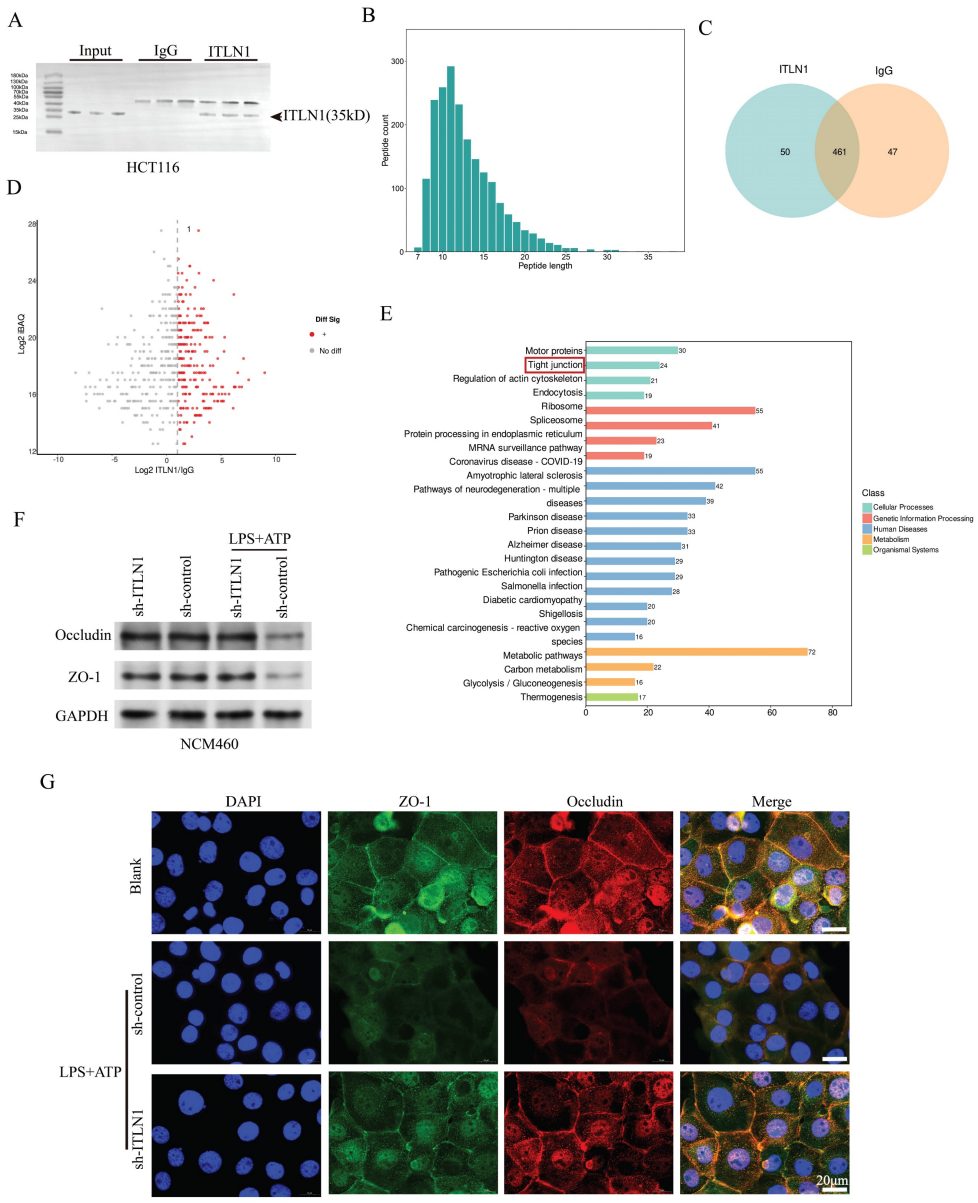
ITLN1 influences tight junctions in IECs. (A) Western blotting analysis following ITLN1 immunoprecipitation, confirming the presence of ITLN1 in the immunoprecipitated complex. (B) Distribution of peptide lengths obtained from mass spectrometry after immunoprecipitation, illustrating the size distribution of peptides associated with ITLN1. (C) Venn diagram displaying the overlap of proteins between ITLN1 and IgG immunoprecipitations. (D) Volcano plot of differentially expressed proteins (threshold: FC > 2, *P <* 0.05). (E) KEGG pathway analysis of differentially expressed proteins. (F) Western blotting analysis illustrating the effect of sh-ITLN1 on the expression of tight junction proteins (occludin and ZO-1) in NCM460 cells with or without LPS/ATP. (G) Immunofluorescence images illustrating the localization and expression of tight junction proteins (occludin and ZO-1) in IECs, with the impact of sh-ITLN1 treatment, indicating disruption of tight junction integrity. Scale bar: 20 μm. CD, Crohn's disease; IECs, intestinal epithelial cells; ITLN1, intelectin-1; DEGs, differentially expressed genes; LPS, lipopolysaccharide; ATP, adenosine triphosphate; FC, fold change; FCM, flow cytometry; KEGG, Kyoto encyclopedia of genes and genomes. Data are presented as mean ± SD. ** *P <* 0.01, *** *P <* 0.001, **** *P <* 0.0001 by one-way ANOVA test.

ITLN1, a galactofuranose-binding secretory lectin, primarily localizes to gastrointestinal goblet cells and omentum 38. To investigate the biological functions and molecular mechanism of ITLN1, we performed IP and MS (Figures 5A-C). Volcano plot analysis highlighted differentially precipitated proteins (Figure 5D), and KEGG pathway enrichment ranked "tight junction" as a top pathway under "cellular processes" (Figure 5E). Previous studies have revealed that ITLN1 can recognize and clear pathogens by binding to specific carbohydrate structures 39 and contribute to the protection of the colonic mucosa 40. Accordingly, we further investigated the impacts of ITLN1 on tight junctions in IECs. Under LPS + ATP-induced inflammation, Western blotting (Figure 5F) and immunofluorescence (Figure 5G) revealed significant downregulation of occludin and ZO-1 in NCM460 cells, which was reversed by sh-ITLN1 knockdown.

### ITLN1 regulates calpain-2 (CAPN2) ubiquitination through competitive binding with tripartite motif containing 8 (TRIM8)

**Figure 6:**
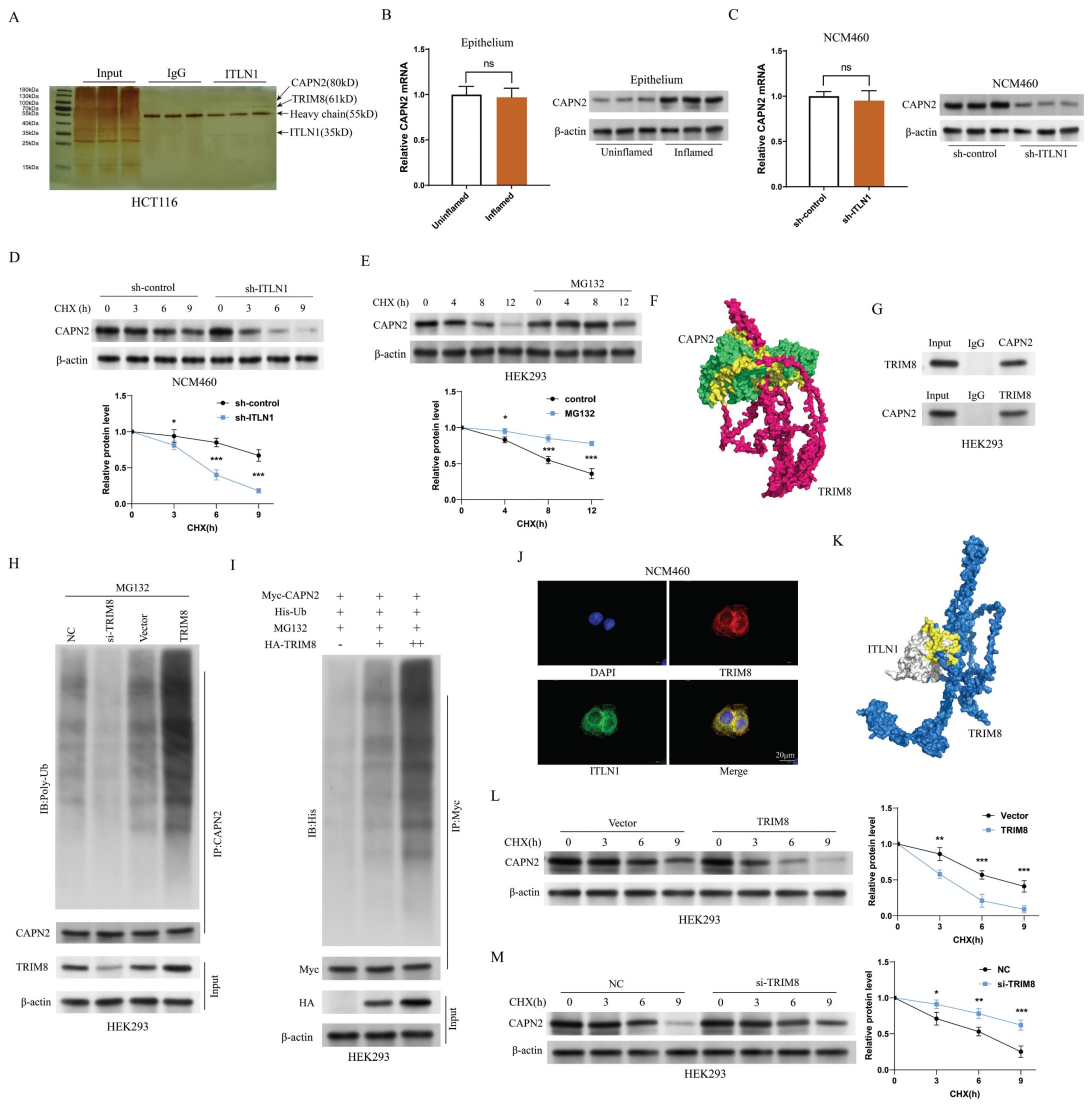
ITLN1 regulates CAPN2 ubiquitination through competitive binding with TRIM8. (A) Silver staining analysis of ITLN1 IP, identifying interacting proteins. (B) mRNA and protein expression levels of CAPN2 in isolated IECs from inflamed and uninflamed colon tissues illustrating no significant change. (C) Impact of sh-ITLN1 on CAPN2 mRNA and protein expression levels in NCM460 cell lines. (D) CHX protein stability assay evaluating the impact of ITLN1 on the stability of CAPN2 protein over time in NCM460 cells. (E) CHX protein stability assay evaluating the stability of CAPN2 protein over time in HEK293 cells. (F) Computational molecular docking analysis identifying potential binding sites between TRIM8 and CAPN2. (G) Bidirectional Co-IP between CAPN2 and TRIM8, confirming their direct interaction. (H) Endogenous and (I) exogenous assessments of TRIM8's impact on the ubiquitination level of CAPN2 protein in HEK293 cells using ubiquitin immunoblotting. (J) Immunofluorescence co-localization of ITLN1 and TRIM8 in NCM460 cells, confirming their spatial proximity. (K) Computational molecular docking analysis revealing potential binding sites between TRIM8 and ITLN1. (L-M) CHX protein stability assay evaluating the impact of TRIM8 and si-TRIM8 on the degradation of the CAPN2 protein in HEK293 cells. IECs, intestinal epithelial cells; IP, immunoprecipitation; ITLN1, intelectin-1; CAPN2, calpain-2; TRIM8, tripartite motif containing 8; CHX, cycloheximide. Data are presented as mean ± SD. ** *P <* 0.01, *** *P <* 0.001, **** *P <* 0.0001 by unpaired t test.

Immunoprecipitation (IP) and mass spectrometry (MS) analyses identified CAPN2 as a primary interactor of ITLN1 (Figure 6A). CAPN2, a calcium-dependent protease, directly participates in apoptosis and necroptosis signaling pathways, making it directly relevant to PANoptosis 41,42.

While CAPN2 mRNA levels remained unchanged between inflamed and non-inflamed intestinal epithelial cells (IECs), its protein expression was markedly upregulated in inflamed regions (Figure 6B). Furthermore, the sh-ITLN1 intervention did not significantly affect CAPN2 mRNA levels but diminished its protein levels (Figure 6C). This suggests that increased protein stability is the main reason for CAPN2 upregulation under inflammatory conditions, with ITLN1 positively regulating its expression by affecting protein stability. The CHX protein stability assay results indicated that MG132 significantly increased CAPN2 protein stability (Figure 6D), suggesting that CAPN2 is mainly degraded via the ubiquitin-proteasome pathway. We re-examined the ITLN1 protein spectrum and found that TRIM8—an E3 ubiquitin ligase—was prominently listed among the interacting proteins (Figure 6A). Computational molecular docking results suggest potential binding sites between TRIM8 and CAPN2 (Figure 6E). Bidirectional Co-IP experiments confirmed that CAPN2 and TRIM8 can bind to each other (Figure 6F). Endogenous Co-IP (Figure 6G) experiments demonstrated that si-TRIM8 (Supplemental Figure 1C) could inhibit the ubiquitination level of the CAPN2 protein, while oe-TRIM8 could upregulate its ubiquitination level. Exogenous Co-IP experiments also confirmed that TRIM8 can induce dose-dependent ubiquitination modification of the CAPN2 protein (Figure 6H). Immunofluorescence results revealed that TRIM8 and ITLN1 were highly co-localized in the NCM460 cell line (Figure 6I). Besides, computational molecular docking suggested potential binding sites between the two proteins (Figure 6J). Additionally, CHX experiments demonstrated that TRIM8 can promote the degradation of the CAPN2 protein (Figure 6K), while si-TRIM8 can inhibit its degradation (Figure 6L). All these results strongly suggest that ITLN1 regulates CAPN2 ubiquitination by competitively binding with TRIM8.

### TRIM8 interacts with CAPN2

**Figure 7:**
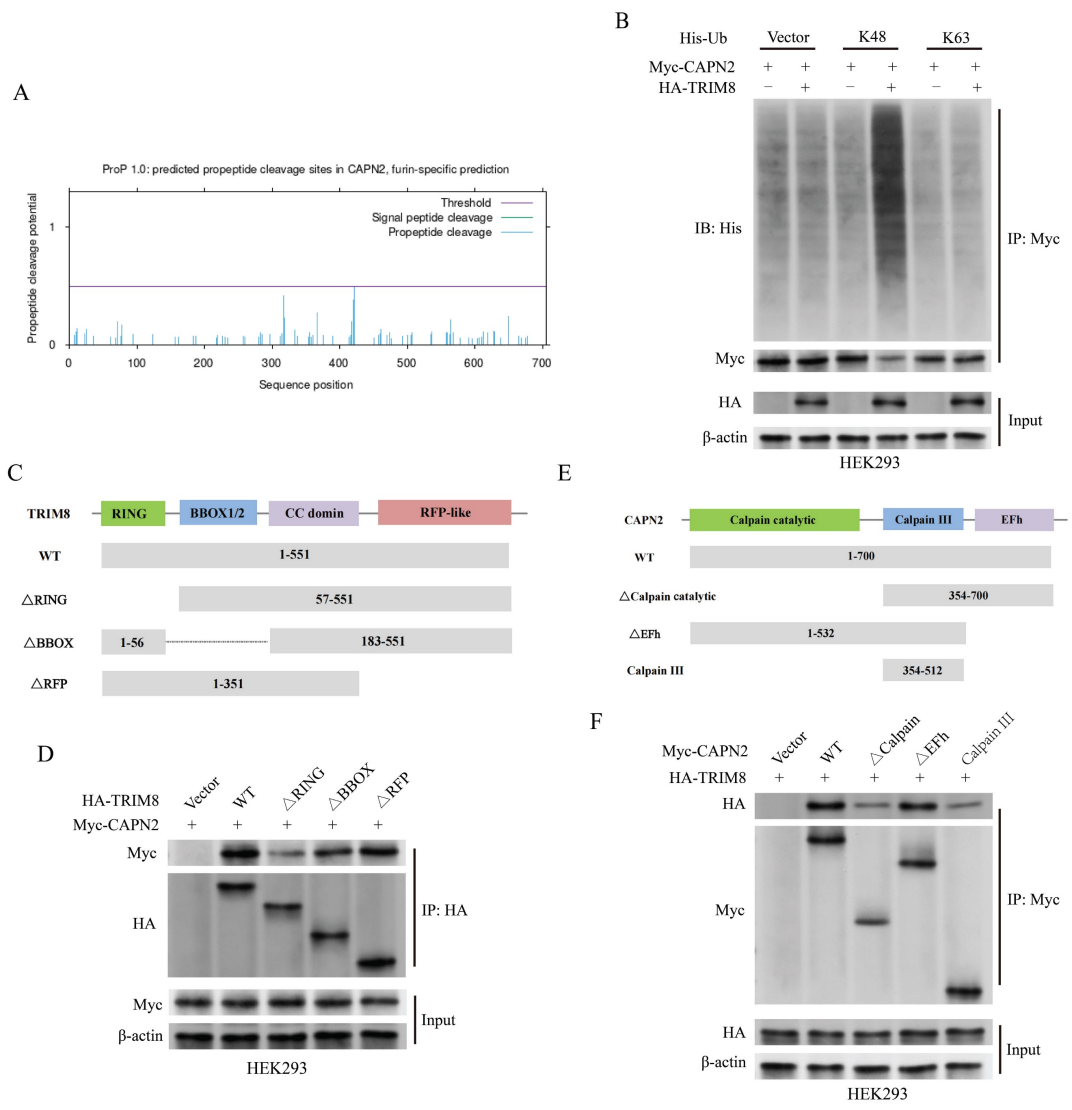
CAPN2 interacts with TRIM8. (A) The predicted ubiquitination sites of CAPN2 determined by Prop1.0, illustrating potential propeptide cleavage regions. (B) Immunoblot analysis of lysates from HEK293 cells transfected with His-tagged K48-Ub or K63-Ub, indicating the ubiquitination pattern of Myc-tagged CAPN2 in the presence or absence of HA-tagged TRIM8. (C) Schematic diagram of TRIM8 and its truncation mutants. (D) Immunoprecipitation of HA-tagged TRIM8 or its mutants and Myc-tagged CAPN2 in HEK293 cells, followed by immunoblotting with the indicated antibodies to analyze their interaction. (E) Schematic diagram of CAPN2 and its truncation mutants. (F) Immunoprecipitation of Myc-tagged CAPN2 or its mutants and HA-tagged TRIM8 in HEK293 cells, followed by immunoblotting to assess the interaction between the proteins.

Figure 7A illustrates the predicted ubiquitination sites of CAPN2 through the database Prop 1.0 43. To characterize TRIM8-mediated polyubiquitination, ubiquitin mutants (K48 and K63) were co-expressed in transfection assays. TRIM8-driven CAPN2 polyubiquitination was observed exclusively with K48 mutants, not K63 (Figure 7B). Structural analysis revealed TRIM8's functional domains: an N-terminal RING-finger domain (essential for E3 ligase activity), BBOX1/2 motifs, a coiled-coil domain, and an RFP-like domain (Figure 7C). Truncated TRIM8 and CAPN2 mutants were constructed to search for the domains responsible for the interaction between CAPN2 and TRIM8. The complementary binding studies using truncated TRIM8 constructs demonstrated that CAPN2 mainly interacted with RING and BBOX domains of TRIM8 (Figure 7D). Moreover, TRIM8 mainly interacted with the calpain catalytic domain of CAPN2 (Figures 7E-F).

### CAPN2 antagonizes the impact of ITLN1 on inflammation and ZBP1-induced PANoptosis

**Figure 8:**
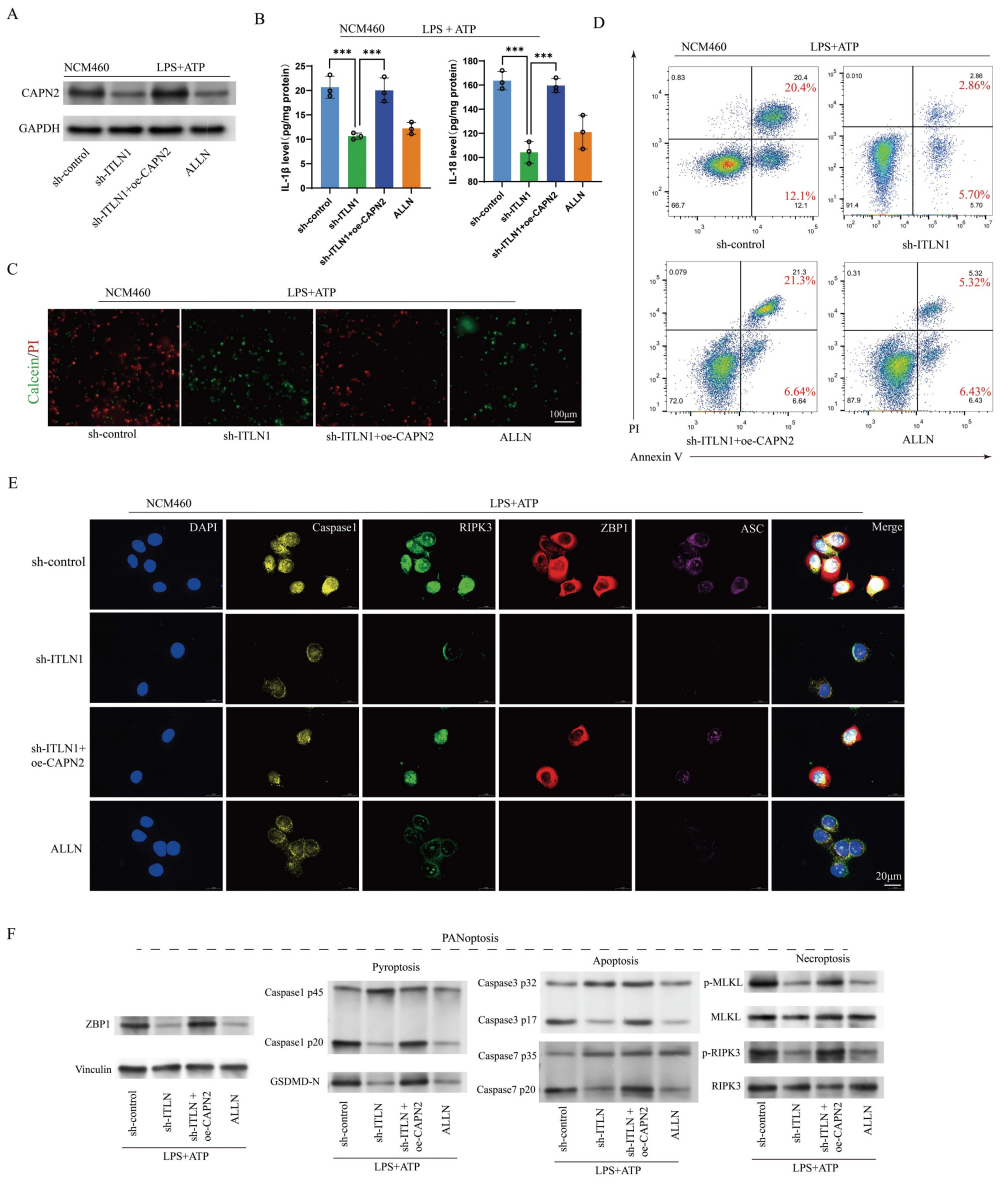
CAPN2 antagonizes the effects of ITLN1 on inflammation and ZBP1-induced PANoptosis. (A) Western blotting analysis illustrating the effect of sh-ITLN1 and CAPN2 rescue on CAPN2 protein expression in NCM460 cells, confirming the modulation of CAPN2 expression upon sh-ITLN1 and CAPN2 rescue treatment. (B) ELISA quantification of proinflammatory cytokines (IL-6 and IL-8) in NCM460 cells, demonstrating the influence of sh-ITLN1 and CAPN2 rescue on cytokine levels following LPS/ATP co-stimulation. (C) Live/dead (Calcein/PI) staining of NCM460 cells indicating the effect of sh-ITLN1 and CAPN2 rescue on cell viability. Scale bar: 100 μm. (D) Flow cytometry analysis of apoptosis in LPS/ATP co-stimulated NCM460 cells treated with sh-ITLN1, sh-ITLN1 + CAPN2 rescue, or ALLN. (E) Immunofluorescence analysis of PANoptosis-related marker genes (caspase-1, RIPK3, ZBP1, and ASC) in NCM460 cells, illustrating the effect of sh-ITLN1 and CAPN2 rescue on PANoptosis expression. Scale bar: 20 μm. (F) Western blotting analysis of PANoptosis-related proteins (caspase-1, caspase-3, caspase-7, GSDMD-N, p-MLKL, p-RIPK3) in LPS/ATP co-stimulated NCM460 cells, indicating the modulation of PANoptosis pathways by sh-ITLN1 and CAPN2 rescue. IECs, intestinal epithelial cells; ITLN1, intelectin-1; CAPN2, calpain-2; FCM, flow cytometry; LPS, lipopolysaccharide; ATP, adenosine triphosphate. Data are presented as mean ± SD. *** *P <* 0.001 by one-way ANOVA test.

We further investigated whether the effect of ITLN1 on inflammation and PANoptosis depends on CAPN2. Intervention with sh-ITLN1 (Supplemental Figure 1D) and ALLN (a CAPN2 inhibitor) significantly suppressed CAPN2 protein levels, while oe-CAPN2 rescued CAPN2 expression (Figure 8A). The inhibitory effect of sh-ITLN1 on LPS + ATP-induced inflammatory cytokines was also rescued by oe-CAPN2 (Figure 8B). Live/dead cell staining (Figure 8C) and apoptosis FCM (Figure 8D) similarly revealed that CAPN2 could counteract the inhibitory effect of sh-ITLN1 on cell death. Moreover, the inhibition of ZBP1-induced PANoptosis-related marker proteins by sh-ITLN1 could be reversed by CAPN2 (Figures 8E-F).

### Sh-ITLN1 improves colonic inflammation in IL-10 KO mice

**Figure 9:**
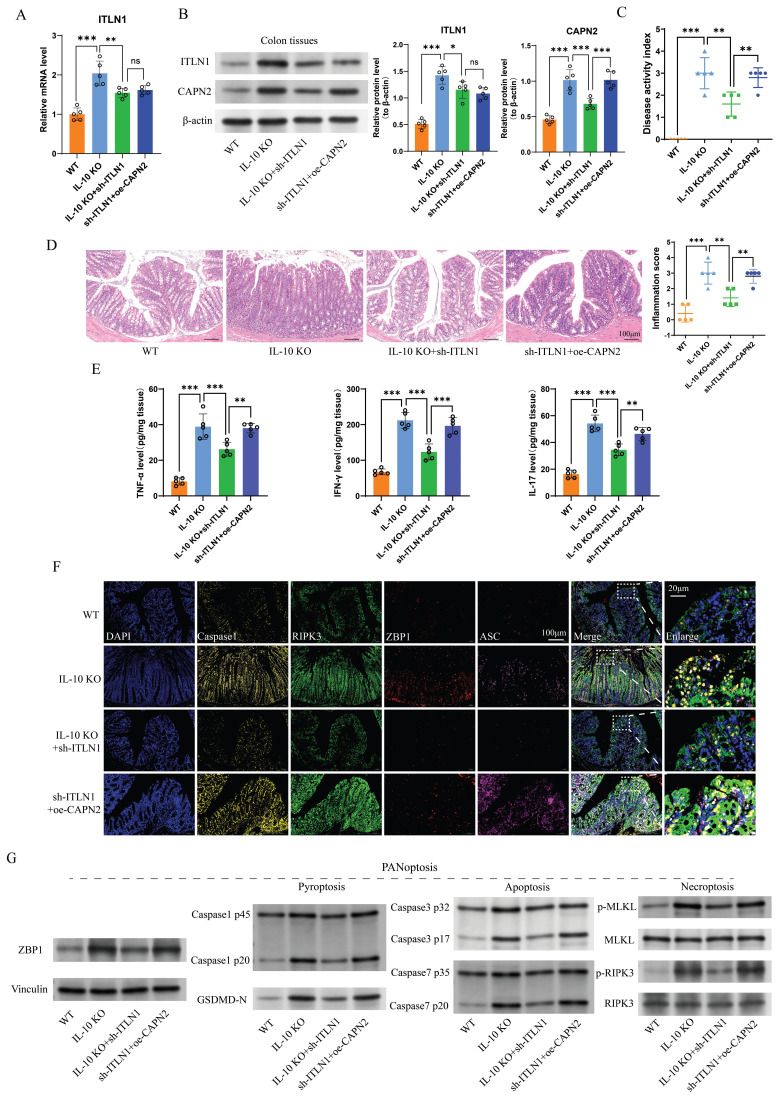
Sh-ITLN1 alleviates colonic inflammation in IL-10 KO mice. (A) Effect of sh-ITLN1 and CAPN2 rescue on ITLN1 mRNA levels in colon tissues of mice, illustrating a significant reduction in ITLN1 expression upon sh-ITLN1 treatment. (B) Western blotting analysis revealing the impact of sh-ITLN1 on ITLN1 and CAPN2 protein expression in colon tissues from IL-10 KO mice. (C) Effect of sh-ITLN1 on the disease activity index, indicating a significant reduction in disease severity in the IL-10 KO model upon ITLN1 knockdown. (D) Histological analysis of colon tissues using HE staining, revealing the influence of sh-ITLN1 on the pathological inflammation score. Scale bar: 20 μm. (E) Quantification of inflammatory cytokine levels (TNF-α, IFN-γ, and IL-17) in colon tissues, demonstrating a significant decrease following sh-ITLN1 treatment. (F) Immunofluorescence and (G) Western blotting analysis of PANoptosis-associated marker genes in colon tissues, demonstrating the effect of sh-ITLN1 and CAPN2 rescue on PANoptosis level. Scale bar: 20 μm. ITLN1, intelectin-1; CAPN2, calpain-2; IL-10 KO, IL-10 knock-out. Data are presented as mean ± SD. ** *P <* 0.01, *** *P <* 0.001 by one-way ANOVA test.

IL-10 KO mice were used as a CD colitis model and administered AAV-carried sh-ITLN1. We found that sh-ITLN1 reduced ITLN1 expression (Figure 9A) and decreased CAPN2 protein levels in colon tissue (Figure 9B). The sh-ITLN1 intervention significantly improved the DAI (Figure 9C), pathological inflammation score (disrupted intestinal integrity, reduced inflammatory cell infiltrates in the lamina propria and reduced goblet cells; Figure 9D), and inflammatory cytokine expression (Figure 9E) in IL-10 KO mice, while CAPN2 counteracted the anti-inflammatory effects of sh-ITLN1. Additionally, sh-ITLN1 significantly inhibited the expressions of ZBP1-induced PANoptosis-related marker proteins of colon tissues, which can be reversed by CAPN2 (Figures 9F-G).

### Sh-ITLN1 improves intestinal barrier function

**Figure 10:**
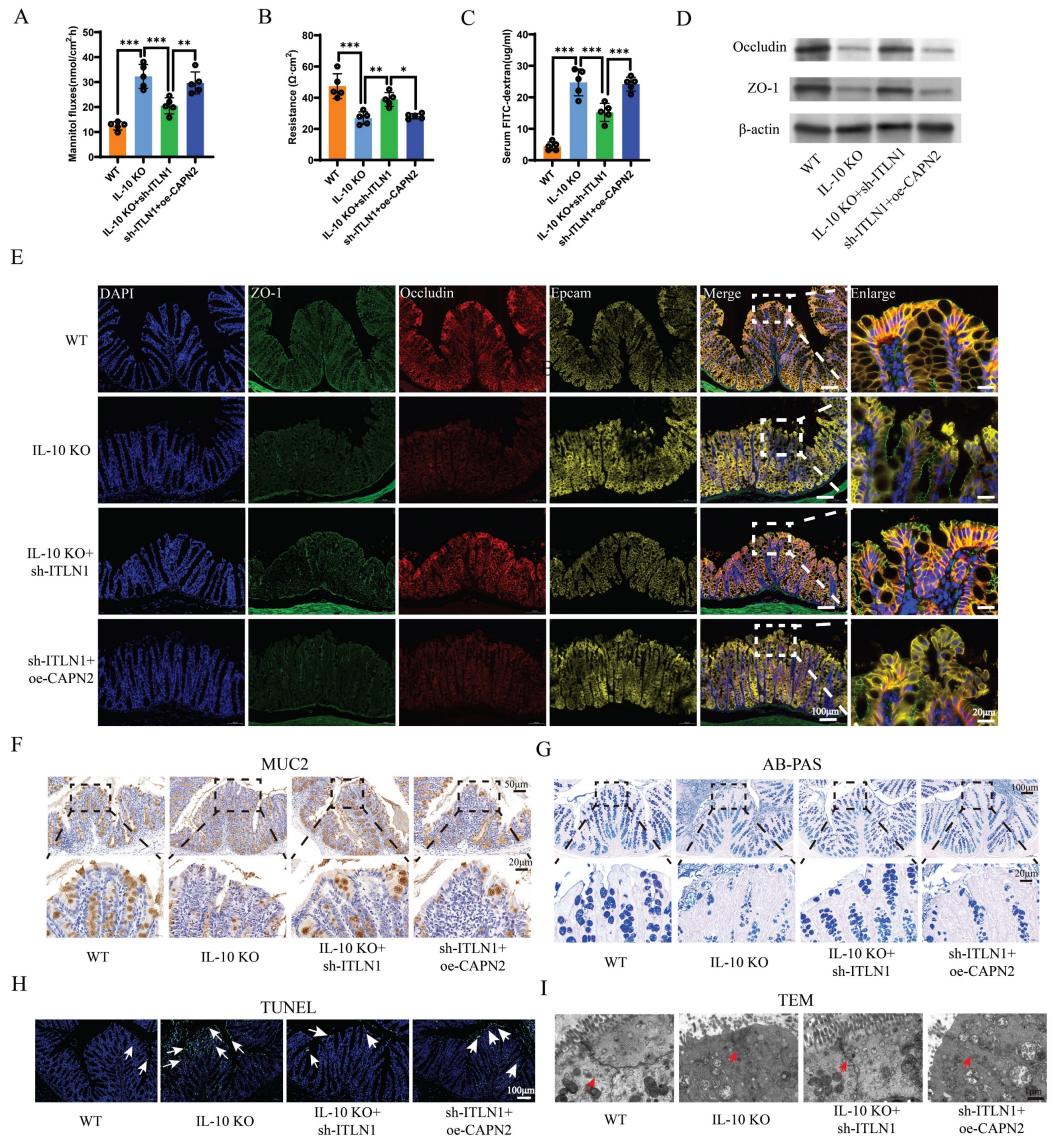
Sh-ITLN1 enhances intestinal barrier function in IL-10 KO mice. (A) Mannitol fluxes, (B) electrical resistance, and (C) FITC-dextran analyses evaluating the effect of sh-ITLN1 and CAPN2 restoration on intestinal permeability. (D) Western blotting and (E) immunofluorescence analysis (scale bar: 100 and 20 μm) illustrating the effect of sh-ITLN1 and CAPN2 rescue on tight junction proteins (occludin and ZO-1) in colon tissues. (F) MUC2 immunohistochemistry in colon tissues. (scale bar: 50 and 20 μm). (G) AB-PAS staining in colon tissues (scale bar: 100 and 20 μm). (H) TUNEL staining in colon tissues (scale bar: 100 μm). (I) Representative TEM images of tight junctions in each group (scale bar: 1 μm). ITLN1, intelectin-1; CAPN2, calpain-2; IL-10 KO, IL-10 knockout; FITC, fluorescein isothiocyanate; TJ, tight junction; AB-PAS, Alcian Blue-Periodic Acid-Schiff; TEM, transmission electron microscope; TUNEL, terminal deoxynucleotidyl transferase dUTP nick end labeling. Data are presented as mean ± SD. * *P <* 0.05, ** *P <* 0.01, *** *P <* 0.001 by one-way ANOVA test.

Sh-ITLN1 treatment significantly improved intestinal permeability in IL-10 KO mice, as evidenced by reduced mannitol flux (Figure 10A), lower FITC-dextran levels (Figure 10C), and increased electrical resistance (Figure 10B). The expression and distribution of tight junction (TJ) proteins, occludin, and ZO-1 were significantly improved with sh-ITLN1 treatment (Figures 10D-E). Similarly, MUC2 immunohistochemistry (Figure 10F) and AB-PAS staining (Figure 10G) revealed that sh-ITLN1 enhanced the mucus barrier in mice. Furthermore, sh-ITLN1 significantly reduced epithelial cell apoptosis in colon tissue (Figure 10H) and improved TJ morphology under TEM (Figure 10I). However, the beneficial effects of sh-ITLN1 on intestinal barrier function in IL-10 KO mice were reversed by CAPN2 (Figures 10A-I).

### Key findings

**Figure 11:**
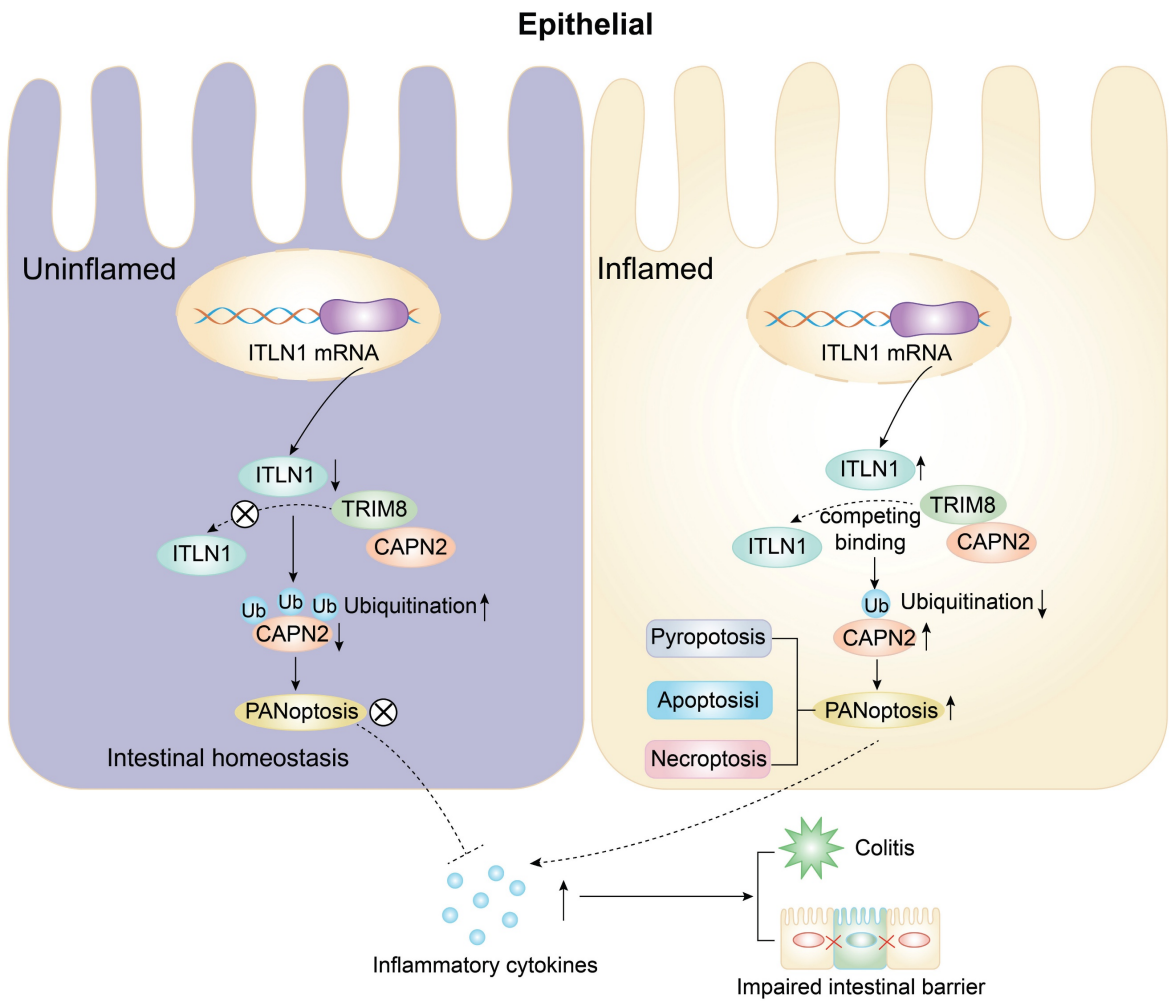
A schematic diagram illustrating how ITLN1 promotes Crohn's colitis by inducing PANoptosis. IECs, intestinal epithelial cells; ITLN1, intelectin-1; CAPN2, calpain-2; TRIM8, tripartite motif containing 8.

The abnormally high expression of ITLN1 competitively binds to TRIM8, inhibiting the ubiquitination of CAPN2 and leading to its increased expression. This induces ZBP1-mediated PANoptosis in IECs, resulting in impaired intestinal mucosal barrier function and the release of a substantial number of inflammatory cytokines, promoting the progression of Crohn's colitis (Figure 11).

## Discussion

Based on the single-cell sequencing,* in vivo,* and *in vitro* studies, this study demonstrated that the ITLN1/TRIM8/CAPN2 axis is involved in the colonic inflammation progression of CD via mediating PANoptosis. This novel insight adds a new dimension to understanding CD pathogenesis and identifies novel clinical therapeutic targets for CD.

Emerging evidence highlights ITLN1's involvement in diverse physiological and pathological processes 44, including inflammation, immune modulation, and metabolic regulation, with a significant association with type 2 diabetes 45. There is some controversy over whether ITLN1 promotes or inhibits inflammation. However, ITLN1's interaction with adiponectin receptor 1 inhibits the NF-κB pathway in macrophages, suppressing LPS-induced proinflammatory cytokine production and demonstrating anti-inflammatory properties 46. Conversely, ITLN1 has been reported to enhance allergen-induced IL-25 and IL-33 secretion in airway epithelial cells, exacerbating allergic airway inflammation 47. Furthermore, ITLN1 has been identified as a potential genetic risk factor for IBD, with significantly elevated expression in ulcerative colitis (UC), possibly participating in UC pathogenesis by disrupting the intestinal mucus barrier and microbiota homeostasis 48. However, the correlation between ITLN1 and CD remains unknown. The mRNA and protein levels of ITLN1 were significantly elevated in IECs in inflamed regions, and its expression was positively correlated with CD-specific inflammatory markers such as CRP, CDAI, and SES-CD. This suggests that there may be a specific correlation between ITLN1 and CD.

CAPN2 is a calcium-dependent cysteine protease involved in various cellular processes. Extensive research has demonstrated a strong connection between CAPN2 and inflammation. A previous study revealed that CAPN1 or CAPN2 depletion provided protective effects against lung inflammation caused by ventilators 49.

Additionally, calpain activation has been linked to neuronal apoptosis following spinal cord injuries and in neurodegenerative disorders 50, highlighting its dual role in inflammatory and degenerative pathologies. Another study has indicated that CAPN1/CAPN2 can facilitate cisplatin-induced pyroptosis in esophageal cancer 51. Similarly, Bozym et al. 52 reported that CAPN2 mediates coxsackievirus B-induced cellular necrosis and is implicated in the actin cytoskeleton rearrangement and disruption of the junctional complex. These findings cumulatively suggest a close relationship between CAPN2 and cell death. Our study has found a positive correlation between CAPN2 and PANoptosis. A previous study has demonstrated the therapeutic potential of CAPN2 inhibitors in murine colitis and colitis-associated cancer, utilizing the azoxymethane/dextran sulfate sodium model to suppress macrophage activation and restrict tumor progression 53. Our findings are consistent with and further support these observations.

Recent research has increasingly highlighted the strong association between PANoptosis and IBD. The excessive IEC death induced by PANoptosis compromises the intestinal barrier, facilitates bacterial translocation, and triggers secondary inflammation, further aggravating mucosal epithelial damage in ulcerative colitis (UC) patients 54,55. Currently, researchers have studied PANoptosis-related genes in CD through a combination of bioinformatics, machine learning, and related experiments. They discovered that PANoptosis plays a nonnegligible role in CD via interacting with CD-associated genes and regulating the immune system 56. Besides, numerous studies have confirmed the direct positive correlation between PANoptosis and inflammation 57. This study demonstrated that the expressions of PANoptosis-related marker proteins were significantly elevated in patients with CD, IL-10 KO colitis mice, and LPS + ATP-induced *in vitro* inflammation models, which strongly suggested the presence of PANoptosis under inflammatory conditions. Furthermore, it revealed that PANoptosis is probably involved in CD pathogenesis, and targeting PANoptosis could become a novel therapeutic modality for CD.

Herein, CAPN2 is identified as one of the key mechanisms by which ITLN1 regulates PANoptosis. ITLN1 does not directly affect CAPN2 mRNA levels; instead, it regulates the protein stability of CAPN2. Protein translational modifications (PTMs) are currently among the most extensively studied mechanisms that regulate protein stability 58. PTMs enhance the functional diversity of the proteome by adding functional groups, regulating subunit proteolysis, or degrading proteins. These modifications include phosphorylation, ubiquitination, methylation, acetylation, and glycosylation 58. Our findings suggest that CAPN2 is primarily degraded via the ubiquitin-proteasome pathway. TRIM8, as an E3 ubiquitin ligase, transfers ubiquitin molecules to target proteins, thereby mediating ubiquitination. This is a crucial mechanism for regulating protein expression levels within cells, impacting key biological processes (autophagy, apoptosis, innate immunity, signal transduction, and others) 59,60. We speculated that ITLN1 may competitively bind to the E3 ligase TRIM8, thereby reducing the ubiquitination level of the CAPN2 protein and promoting its increased expression.

This study elucidates a pathogenic pathway in which ITLN1 competitively binds TRIM8 to inhibit CAPN2 ubiquitination and degradation, stabilizing CAPN2 to promote ZBP1-mediated PANoptosis in IECs and exacerbate Crohn's colitis. This mechanism offers viable therapeutic options: targeted inhibition of ITLN1 (via shRNA or monoclonal antibodies) or pharmacological inhibition of CAPN2 (using calpain inhibitors such as MDL28170) significantly reduced intestinal inflammation and PANoptosis *in vitro* and *in vivo.* These effects were accompanied by the restoration of mucosal barrier integrity in colitis models. This establishes a robust basis for subsequent clinical translation. Targeting the ITLN1/CAPN2 axis reduced inflammation and PANoptosis significantly and restored mucosal barrier integrity in colitis models, thereby establishing a basis for subsequent clinical application. This corresponds with other therapeutic strategies in IBD, including polyphenols, which regulate inflammation and gut microbiota, enhancing barrier function 61. We intend to validate ITLN1-neutralizing biologics in patient-derived organoids and evaluate ITLN1 as a biomarker for anti-PANoptosis therapies, bridging mechanistic insights to precision treatments in CD.

## Supplementary Material

Supplementary figure S1.

## Figures and Tables

**Table 1:** Inclusion and exclusion criteria

Inclusion criteria
aged 18-65 years
diagnosed with CD based on pathological evidence
underwent elective surgical resection for colonic (or ileocolonic) CD
with signed informed consent
**Exclusion criteria**
with diffuse small bowel lesions
combined with malignancies
combined with other autoimmune or autoinflammatory diseases
declined to participate

CD, Crohn's disease.

**Table 2:** Patient demographic data

	Single-cell sequencing (Discovery set)	Validation set
Number	4	10
Age (year)	42.0±2.2	42.3±5.3
Gender, n (%)	-	-
Male	3 (75.0)	7 (70.0)
Female	1 (25.0)	3 (30.0)
Body mass index (kg/m^2^)	20.9±0.9	21.2±1.2
Disease location, n (%)	-	-
Colonic	2 (50.0)	3 (30.0)
Ileocolic	2 (50.0)	7 (70.0)
Duration of disease (month)	21.3±6.5	21.8±6.3

Data are expressed as means ± S.E.M or number with percentage.

**Table 3:** Primers sequences for qRT-PCR

Name	Sequence (5′-3′)
Human IGHG3	
Forward	CTCTACTCCCTCAGCAGCGTG
Reverse	CTGGGCTTGTGATTCACGTT
Human ITLN1	
Forward	AGTGTTGGACTGACAACGGC
Reverse	TACATCCGGTGACCCTCATTC
Human HMGCS2	
Forward	GCCTCTCAGGACATGTTCGAC
Reverse	AGCCATAAGAGAAGGCACCA
Human CAPN2	
Forward	CTCCACCAAGTCATCGTTGCT
Reverse	TTCCAGTATTCTCGGGATCCAG
Human TRIM8	
Forward	CGGAGAATTGGAAGAACTGCT
Reverse	TGTAGGCCTGGTTGCACTCTG
Human GAPDH	
Forward	TCCTGGGCTACACTGAGCAC
Reverse	CTGTTGCTGTAGCCAAATTCGTTG
Mouse ITLN1	
Forward	ACTCACAATGGGTACAGCAGTAG
Reverse	CATGCCTTGGAGCCCACAATG
Mouse TRIM8	
Forward	TGCGGAAGATGCTAGAAGGTC
Reverse	GTCTCCAGGAAGCTAGCCTCA
Mouse CAPN2	
Forward	GACATGCACACCATTGGCTT
Reverse	CGCGGAGGTTAATGAAGGTAT
Mouse GAPDH	
Forward	GTCAAGGCCGAGAATGGGAA
Reverse	CTCGTGGTTCACACCCATCA

qRT-PCR, quantitative real time-polymerase chain reaction; ITLN1, intelectin-1; CAPN2, calpain-2; TRIM8, tripartite motif containing 8; IGHG3, immunoglobulin heavy constant gamma 3; HMGCS2, 3-hydroxy-3-methylglutaryl-CoA synthase 2.

**Table 4:** Primers for sh- and si-RNA sequences

Name	Sequence (5′-3′)
Human sh-ITLN1-1	Target: GCTAATACTTACTTCAAGGAA
Forward	CCGGGCTAATACTTACTTCAAGGAACTCGAGTTCCTTGAAGTAAGTATTAGCTTTTTG
Reverse	AATTCAAAAAGCTAATACTTACTTCAAGGAACTCGAGTTCCTTGAAGTAAGTATTAGC
Human sh-ITLN1-2	Target: GATATGGAACTCATGTTGGTT
Forward	CCGGGATATGGAACTCATGTTGGTTCTCGAGAACCAACATGAGTTCCATATCTTTTTG
Reverse	AATTCAAAAAGATATGGAACTCATGTTGGTTCTCGAGAACCAACATGAGTTCCATATC
Human sh-ITLN1-3	Target:CCAGTGAAATATGGAGAAGGA
Forward	CCGGCCAGTGAAATATGGAGAAGGACTCGAGTCCTTCTCCATATTTCACTGGTTTTTG
Reverse	AATTCAAAAACCAGTGAAATATGGAGAAGGACTCGAGTCCTTCTCCATATTTCACTGG
Human si-TRIM8-1	Target: GCCAGTACTGCTGCTACTACAGC
Forward	UGUAGUAGCAGCAGUACUGGC
Reverse	CAGUACUGCUGCUACUACAGC
Human si-TRIM8-2	Target: AGGATGTCAGCTTCATGAAGAAC
Forward	UCUUCAUGAAGCUGACAUCCU
Reverse	GAUGUCAGCUUCAUGAAGAAC
Human si-TRIM8-3	Target: CACCAAGTCTGTGAAAATCCTGA
Forward	AGGAUUUUCACAGACUUGGUG
Reverse	CCAAGUCUGUGAAAAUCCUGA
Mouse sh-ITLN1-1	Target: CTCGGAAGACAGCCTCTTATT
Forward	CCGG CTCGGAAGACAGCCTCTTATT CTCGAG AATAAGAGGCTGTCTTCCGAG TTTTTG
Reverse	AATTCAAAAA CTCGGAAGACAGCCTCTTATT CTCGAG AATAAGAGGCTGTCTTCCGAG
Mouse sh-ITLN1-2	Target: CTCGGAAGACAGCCTCTTATT
Forward	AATTCAAAAA CTCGGAAGACAGCCTCTTATT CTCGAG AATAAGAGGCTGTCTTCCGAG
Reverse	AATTCAAAAA CCAGCATTACCTGTAGTCTAT CTCGAG ATAGACTACAGGTAATGCTGG
Mouse sh-ITLN1-3	Target:CTCGGAAGACAGCCTCTTATT
Forward	CCGG CACGAAGAATGGTGTCATCTA CTCGAG TAGATGACACCATTCTTCGTG TTTTTG
Reverse	AATTCAAAAA CACGAAGAATGGTGTCATCTA CTCGAG TAGATGACACCATTCTTCGTG

qRT-PCR, quantitative real time-polymerase chain reaction; ITLN1, intelectin-1; CAPN2, calpain-2.
