# Supplementary material for: ITLN1 exacerbates Crohn's colitis by driving ZBP1-dependent PANoptosis in intestinal epithelial cells through antagonizing TRIM8-mediated CAPN2 ubiquitination
Source: Int J Biol Sci. 2025 May 31;21(8):3705–25. doi: 10.7150/ijbs.105550 (PMC12160931; doi:10.7150/ijbs.105550)

Supplemental Figure 1: Validation of knockdown efficiency for sh-/si-RNA.

(A) Evaluation of sh-ITLN1 efficacy in NCM460 cells by qRT-PCR, and (B) Western blotting.

(C) Evaluation of si-TRIM8 efficacy in NCM460 cells by qRT-PCR.

(D) Evaluation of sh-ITLN1 efficacy in colon tissues of IL-10 KO mice.

ITLN1, intelectin-1; TRIM8, tripartite motif containing 8; IL-10 KO, IL-10 knock-out.

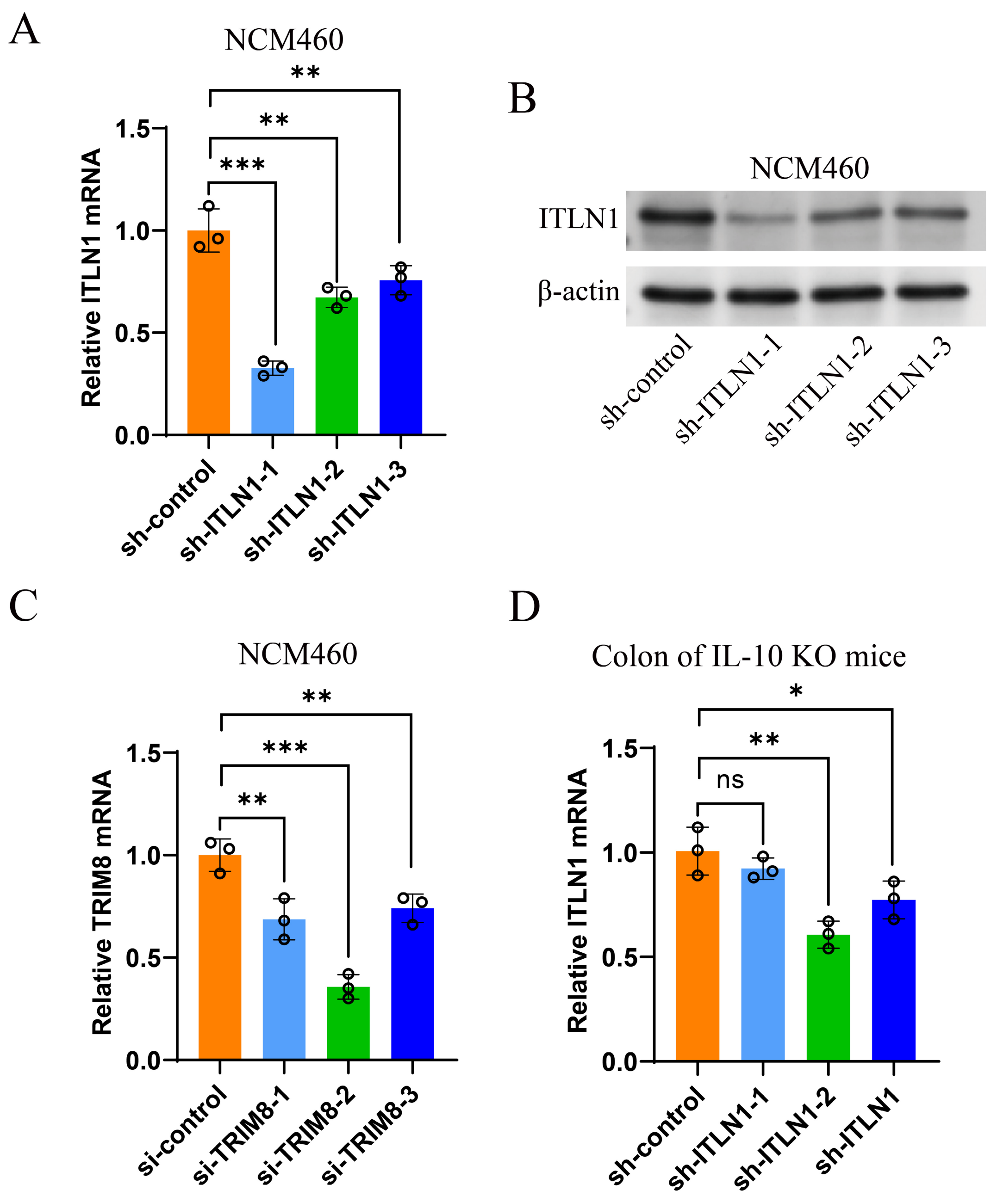

Supplement: Supplementary file 1 — Supplementary figure S1. [file ijbsv21p3705s1.pdf]
